# Comparison of the efficacy of the convex side short fusion combined with concave side single growing rod technique and the traditional bilateral growing rod technique in the treatment of early onset scoliosis

**DOI:** 10.1186/s12891-024-07457-3

**Published:** 2024-04-26

**Authors:** Weiwei Zhu, Xuejun Zhang, Jun Cao, Baihui Zhang, Wenhao Chen, Yunsong Bai, Dong Guo, Ziming Yao

**Affiliations:** 1 Children’s Hospital, Zhejiang University School of Medicine, National Clinical Research Center for Child Health, Hangzhou, China; 2 Beijing Children’s Hospital, Capital Medical University, National Children’s Medical Center, Beijing, China

**Keywords:** Early Onset Scoliosis, Growing Rod Technique, Efficacy

## Abstract

**Objectives:**

The application of a growing rod technique can retain the growth and development potential of the spine and thorax while controlling the progression of scoliosis deformity. Theoretically, convex side short fusion combined with a concave side single growing rod technique can significantly reduce the asymmetric growth of the spine in the vertex region in most patients. However, the final clinical outcome of various techniques is yet to be clearly determined and compared between studies. Therefore, we compared the efficacy of these two growing rod techniques in treating early onset scoliosis.

**Methods:**

In a retrospective study of 152 EOS patients seen between 2013.1 and 2019.12, 36 cases of EOS patients were selected for inclusion. Among the 36 cases, 11 cases were treated with convex side short fusion combined with a concave side single growing rod technique, group (A) The remaining 25 cases were treated with traditional bilateral growing rod technique, group (B) Age, gender, etiology, follow-up time, Cobb angle of main curve, T1-S1 height, coronal trunk shift, sagittal vertical axis (SVA), Cobb angle of thoracic kyphosis at last follow-up, and Cobb angle at proximal junction kyphosis of the first and last post-operation follow-up were recorded. In addition, internal fixation related complications, infection, nervous system complications were recorded as well.

**Results:**

There was no statistically significant difference between group A and group B in preoperative age, Cobb angle of main curve, coronal trunk shift, T1-S1 height, SVA, Cobb angle of thoracic kyphosis (*p* > 0.05). However, at the last follow-up (Group A, mean 4.4 ± 1.01 years; Group B, mean 3.6 ± 0.01 years) the Cobb angle of the main curve was less and T1-S1 height greater in group A compared with group B (*p* < 0.05). There was no statistically significant difference between group A and group B in the correction rate of the Cobb angle of the main curve or the growth rate of T1-S1 height (*p* > 0.05). There was no statistically significant difference in the coronal imbalance ratio, thoracic kyphosis abnormality ratio, or the occurrence PJK ratio between group A and group B at the last follow-up (*p* > 0.05), but the sagittal imbalance ratio and internal fixation abnormality ratio were higher in group A than in the group B (*p* < 0.05).

**Conclusions:**

During the treatment of EOS, both the convex side short fusion combined with concave side single growing rod technique and traditional bilateral growing rod technique can correct the Cobb angle of main curve with no significant hindering of the spinal growth observed. The traditional bilateral growing rod technique has advantages in control of the sagittal balance of the spine, and the complications associated with internal fixation were lower.

## Background

Treating Early Onset Scoliosis (EOS) is difficult, and it is a controversial topic in the treatment of children’s spinal deformities. Nowadays, when conservative treatment measures such as brace and sequential cast are ineffective, the main surgical treatment of EOS is the growing rod techniques. The most widely used growing rod techniques in the world are the magnetically controlled growing rod technique, traditional (double or single) growing rod technique, convex side block combined with concave side single growing rod technique, osteotomy with short segmental fusion, and the dual growing rod technique [1–3]. Comparative studies on the treatment of different growing rod techniques have been reported in the literature [4–7]. The complications of traditional bilateral growing rod technique are less than those of single growth rod; The number of reoperation incision times in the magnic growing rod technique is lower than that of traditional bilateral growing rod, but the complications related to internal fixation are still high, and the device materials still need to be further improved; The traditional bilateral growing rod combined with apic area osteotomy internal fixation is gradually used in the treatment of early-onset scoliosis with severe deformity [1–7]. However, some special children in clinical practice cannot tolerate the placement of bilateral growing rods, and the treatment of such patients is still a challenge.There is a gap in the literature about the outcome between convex side short fusion combined with concave side single growing rod technique and traditional dual growing rod technique in the treatment of EOS.The structural stability of the spine after unilateral short segment fixation remains unknown; it is also unknown whether the unilateral fixation screw requires multiple replacement in subsequent lengthening procedures, and whether this technique allows longitudinal growth of the spine while exerting control over deformities in the vertevertebral region as expected.Therefore, we aimed to compare the effect and complications of these two growing rod techniques in the treatment of EOS.

## Methods

### General information

Inclusion Criteria: (1) Diagnosis of EOS; (2) Using convex side short fusion combined with concave side single growing rod technique or traditional dual growing rod technique; (3) Follow-up time ≥ 3 years; (4) Complete follow-up imaging data; (5) All anchors were pedicle screws.

Exclusion Criteria: (1) History of other spinal internal fixation; (2) History of spinal trauma; (3) Only use single growing rod technique.

One hundred and fifty-two (152) different EOS patients were treated between 2013.1 and 2019.12 in our department, and 36 EOS patients were included in this study. Of these, 11 cases were treated with convex side short fusion combined with concave side single growing rod technique (group A) and another 25 cases were treated with traditional dual growing rod technique (group B).

### Observation indicators

Age, gender, etiology, follow-up time, Cobb angle of main curve in preoperative and last follow-up, the height of T1-S1 at preoperative and last follow-up (vertical distance between upper endplate of T1 and upper endplate of S1), cervical 7 plumb line (C7PL) center sacral vertical line (CSVL) at preoperative and last follow-up, Sagittal vertical axis(The horizontal offset from a plumbline dropped from C7 to the postero-superior corner of S1,SVA) at preoperative and last follow-up, thoracic kyphosis at last follow-up (Cobb angle between T5 superior vertebral endplate and T12 inferior vertebral endplate), proximal junctional kyphosis (PJA) of all spine lateral X-ray at first standing and last follow-up, Cobb angle between the lower endplate of the upper end fixation and the upper endplate of the two vertebrae above. Proximal junctional kyphosis (PJK) criteria: (1) PJA > 10°; (2) compared with preoperative, PJA > 10°. Abnormal internal fixation (pedicle screw loosening, displacement, pull-out; PJK, rod breakage, revision) were recorded during the follow-up period. All data were measured independently by a single spinal surgeon. Intra-observer evaluations were performed for each patient three times by another expert.The last follow up in this study refers to the last follow up when the growing rod technique did not experienced growing-rod graduate. However, with the gradual growth of the children, they still have to experience the growing-rod graduation treatment.

### Statistical analysis

Statistical analysis was performed by using SPSS 22.0 software. The measurement data is expressed by mean ± standard deviation, a paired sample t test was used to compare the differences of measurements between patients before and at the last follow-up, and an independent sample t test and chi-square test were used to compare the differences of corresponding measurements between the two groups. A *p* < 0.05 was recognized as statistical significance.

### Surgical technique

Group A children received the one-stage posterior convex side short fusion and concave side single growing rod technique. A standard midline incision was made according to the locations of osteotomy level. Insertion of pedicle screws at the adjacent vertebrae above and below the osteotomy. The vertebral was partly or fully resected from the convex side in children with congenital scoliosis. For other types of early-onset scoliosis, a facet joint resection release was used. After a well-bent unilateral rod was inserted on the convex side, gradual compression was applied. In terms of implantation of the growing rod, the cephalad and caudal anchor sites were exposed, and fixations were placed in two levels. Then, bilateral overlapping rods were tunneled with a rod-to-rod connector in a submuscular manner. Group B patients were treated by placing bilateral rods on both sides in each patient. During the follow-up period, the interval between subsequent lengthening procedures was scheduled for approximately 8 to 12 months.

## Results

### General data of two groups

Group A, nine males and two females, an mean follow-up of 4.4 ± 1.01 years. Etiology: congenital five cases, idiopathic two, neurofibromatosis in three cases, syndrome, one case. Group B, 11 males and 14 women, an mean follow-up of 3.6 ± 0.01 years. Etiology: 13 cases congenital, idiopathic five, neurofibromatosis in six cases, syndrome one case.

**Fig. 1:**
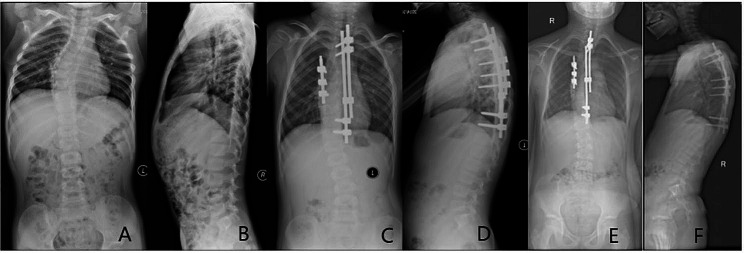
6-year-old boy, syndromic early onset scoliosis. **A**, **B**, Preoperative X-ray; **C**, **D**, use convex side block combined with concave side single growing rod technique, after first operation, X-ray; **E**, **F**, Sixty-eight months (four lengthening) follow up

**Fig. 2:**
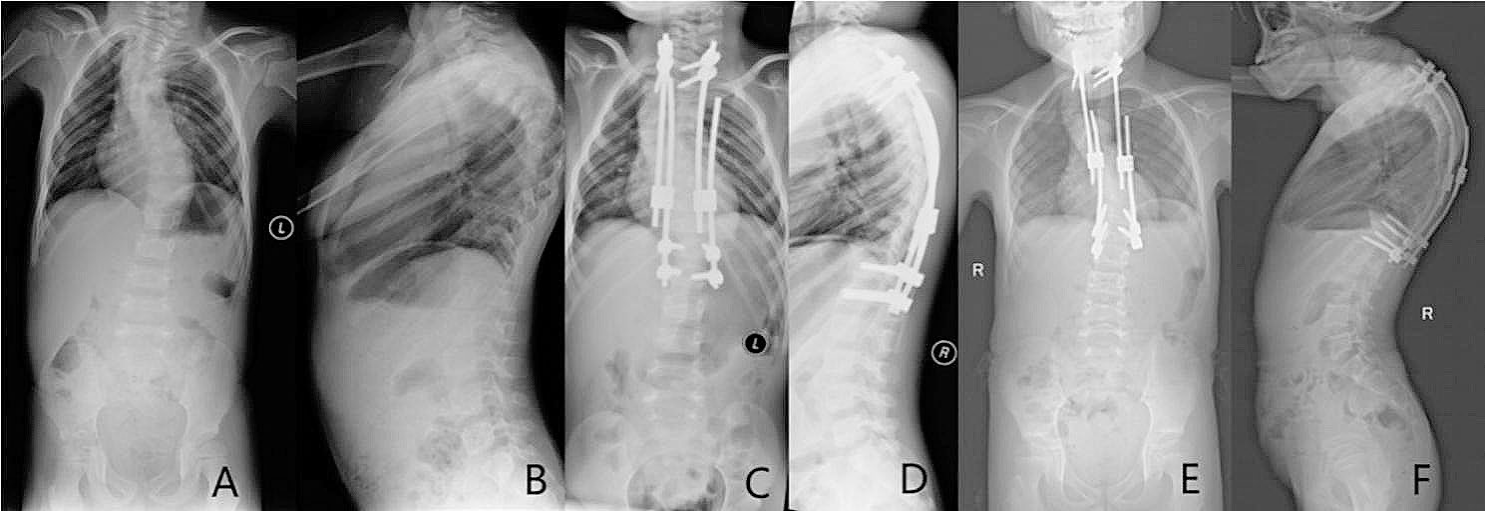
6-year-old girl, syndromic early onset scoliosis; **A**, **B**, Preoperative X-ray; **C**, **D**, Use traditional bilateral growing rod technique, after first operation, X-ray; E, F, Seventy-seven months (six lengthening) follow-up

There was no significant difference between group A and group B in preoperative age, Cobb angle of the preoperative main curve, C7PL-CSVL, T1-S1 height, preoperative thoracic kyphosis Cobb angle, or preoperative SVA (*p* > 0.05). (Table 1; Figs. 1 and 2)

**Table 1:** Preoperative data of group A and B

group	age (years)	cobb angle of main curve(°)	C7PL-CSVL (mm)	T1-S1 height(cm)	preoperative thoracic kyphosis cobb angle(°)	preoperative SVA(mm)
A	5.93 ± 2.90	58.23 ± 20.62	11.74 ± 6.73	27.45 ± 3.70	30.75 ± 14.22	16.99 ± 12.48
B	5.98 ± 2.03	65.7 ± 19.62	12.44 ± 9.75	26.77 ± 4.27	33.77 ± 26.01	23.60 ± 15.32
t value	-0.055	-1.036	-0.218	0.453	-0.36	-1.256
*P value*	0.957	0.307	0.829	0.653	0.721	0.218

### Parameters in each group

For group A, there was a statistically significant difference in the Cobb angle of the main curve and height of T1-S1 before and after the operation (*p* < 0.05). In addition, there was no significant difference in the result of C7PL-CSVL, SVA, and thoracic kyphosis Cobb angle (*p* > 0.05). (Table 2)

**Table 2:** Comparison of preoperative and last follow-up parameters in Group A

parameters	preoperative	last follow-up	t value	P value
cobb angle of main curve(°)	58.23 ± 20.62	42.43 ± 16.52	2.472	0.033
height of T1-S1(cm)	27.45 ± 3.70	31.97 ± 4.05	-3.781	0.004
C7PL-CSVL(mm)	11.74 ± 6.73	14.55 ± 18.28	-0.488	0.636
SVA(mm)	16.99 ± 12.48	34.50 ± 28.48	-1.953	0.079
thoracic kyphosis cobb angle(°)	30.75 ± 14.22	33.41 ± 11.75	-0.415	0.687

For group B, there was a statistically significant difference in the Cobb angle of the main curve, height of T1-S1, and the thoracic kyphosis Cobb angle before and after the operation (*p* < 0.05). The C7PL-CSVL and SVA were not significantly different. (*p* > 0.05). (Table 3)

**Table 3:** Comparison of preoperative and last follow-up parameters in group B

parameters	preoperative	last follow-up	t value	P value
cobb angle of main curve(°)	65.70 ± 19.62	40.18 ± 14.89	8.103	<0.001
height of T1-S1(cm)	26.77 ± 4.27	31.46 ± 4.36	-7.594	<0.001
C7PL-CSVL(mm)	12.44 ± 9.75	14.69 ± 14.32	-0.685	0.500
SVA(mm)	23.60 ± 15.32	25.78 ± 23.42	-0.446	0.660
thoracic kyphosis cobb angle(°)	33.77 ± 26.01	22.70 ± 13.25	2.224	0.036

### Comparison of parameters and complications between the two groups

There was no statistically significant difference between the correction rate of the main curve, T1-S1 height growth rate, C7PL-CSVL change, and SVA change between group A and group B (*p* > 0.05). At the last follow-up measuring thoracic kyphosis the Cobb angle in group B was less than in group A (*p* < 0.05). (Table 4)

**Table 4:** Comparison of Group A and B Parameters

parameters	Group A	Group B	t value	P value
Correction rate of main curve	0.23 ± 0.28	0.37 ± 0.19	-1.703	0.098
T1-S1 height growth rate	0.14 ± 0.11	0.19 ± 0.15	-1.054	0.299
last follow-up C7PL-CSVL (mm)	14.55 ± 18.28	14.69 ± 14.32	-0.025	0.980
last follow-up SVA(mm)	34.50 ± 28.48	25.78 ± 23.42	0.963	0.342
last follow-up thoracic kyphosis(°)	33.41 ± 11.75	22.70 ± 13.25	2.307	0.027

At the last follow-up there was no statistically significant difference between group A and group B in the proportion of coronal imbalance, the proportion of thoracic kyphosis abnormality, and the proportion of PJK (*p* > 0.05). At the last follow-up, the proportion of sagittal imbalance and abnormal internal fixation in group A were significantly higher than in group B (*p* < 0.05). Among them there were seven cases (7/11) of sagittal imbalance in group A (63.6%) and five cases (5/25) of sagittal imbalance in group B (20%). There were four cases of a broken rod (4/11) in group A (36.4%), and one case (1/25) in group B (4%). There were eight cases were the pedicle screw loosened and displacement occurred in group A and six cases occurred in group B. Group A had four cases of renovation where fractured rods were removed, and three cases were renovated with traditional bilateral growth rods. In only one case was the original plant removed, final fusion. There was one case of a broken rod in group B that required the replacement of rods during renovation, prolonging the lower anchor segments, and use of a traditional bilateral growing rod. No screw fracture, no surgical incision infection, no neurological complications, and no deaths occurred in either group. (Table 5)

**Table 5:** Comparison of parameters between group A and B

parameters(number)	group A	group B	P value
Coronal imbalance	3	6	0.571
Coronal balance	8	19	
Sagittal imbalance	7	5	0.020
Sagittal balance	4	20	
Abnormal thoracic kyphosis	3	5	0.678
Normal thoracic kyphosis	8	20	
PJK	2	3	0.631
no PJK	9	22	
Internal fixation anomalies	8	6	0.010
Internal fixation normal	3	19	

## Discussion

### Comparison of design theory

The principle of the traditional bilateral growing rod technique is to use the upper and lower end anchors of the spine and the growth rods in series on both sides to extend the spine longitudinally, which is equivalent to applying traction at the head and tail ends of the scoliosis segment. By means of an adjacent intervertebral disc and vertebral body, the tension is transmitted from the two ends to the middle to correct scoliosis, and spine growth is maintained by the lengthening operation. However, the defect of this technique is that the apex vertebrae area of scoliosis cannot directly exert a strong force. The original intention of a convex side short fusion combined with a concave side single growing rod technique is to fix the convex side of the apex vertebrae area using short segment pedicle screws, directly apply a strong control force, and directly block the growth of the convex side of the apex vertebrae area. At the same time there is an anchor point of the head and tail end of the concave spine and the single growing rod longitudinal extension of the concave side spine. This offers direct control and correction of scoliosis in the apex vertebrae area. The growth of the spine is then realized by the lengthening operation of the concave side growing rod.

Congenital scoliosis in EOS, especially long-segment mixed scoliosis with kyphosis, often progresses rapidly in the apical region. A traditional growing rod technique, even with bilateral growth rods, often make it difficult to achieve satisfactory and prolonged orthopedic control over the progression of apical deformities due to the inherent deficiencies of the aforementioned technical design principles. Therefore, for congenital early scoliosis with a larger kyphosis, some reports have proposed the technique of osteotomy and internal fixation combined with bilateral growth rods [4, 5]. Further studies have pointed out that in the treatment of congenital early scoliosis with a large kyphosis, the technique of osteotomy and pedicle screw internal fixation combined with a bilateral growth rod in the apical area is superior to the traditional bilateral growth rod technique [8].

Convex side short fusion combined with a concave side single growing rod technique is in principle connected with an apical area vertebral osteotomy and pedicle screw internal fixation combined with a bilateral growing rod technique [9]. Yet for EOS children with better flexibility, such as idiopathic EOS, neurofibromatosis EOS, and congenital EOS of long segments, we only use a low-grade osteotomy in the topical vertebrae, such as an articular process joint osteotomy or partial lamina osteotomy. We did not perform a high-grade osteotomy, such as semi-vertebral complete resection, trans-pedicular vertebral wedge osteotomy, or total or subtotal vertebral resection. The pedicle screw and short rod were fixed in 1–2 segments of the convex side of the vertebrae area to block the growth of the convex spine, and the longitudinal extension of the concave spine was carried out with the help of a concave single growing rod.

### Comparison of the outcome

It is confirmed that the single growing rod technique on the concave side combined with a convex side block can effectively correct the main curve and allow the longitudinal growth of the spine. There was no significant difference in the correction rate of the main curve Cobb angle between group A and group B, which indicated that the single growing rod technique on the concave side combined with a convex side block and the traditional bilateral growing rod technique can effectively correct the main curve. The effect of the two techniques to correct the main curve is similar. Notably, there was no significant difference in the T1-S1 height growth rate, and the two techniques allowed the same longitudinal growth of the spine. The convex side block did not have a negative effect on the overall height growth of the T1-S1 spine.

In this study, different types of EOS patients were treated (including congenital, idiopathic, neurofibromatosis, syndrome, etc.) with convex side block combined with concave side single growing rod technique. When focus on the main curve correction, the convex side block combined with concave side single growing technique is a better treatment option than the traditional bilateral growing rod technique. We analyzed that this may be due to the block of unilateral short segment pedicle screw-rod, which can more directly effect the orthopedic force on the vertex of the spinal deformity.The traditional bilateral growing rod technique use indirect force for distraction, through the disc between the upper and lower anchor points, which inevitably leads to the loss of the force ultimately acting on the apex of the deformity.But when focus on the sagittal kyphosis in the spine, traditional bilateral growing rod technique is better than the convex side block combined with concave side single growing technique. This suggests that for EOS patients with a large thoracic kyphosis, the traditional bilateral growing rod technique is more suitable than the convex side block combined with concave side single growing rod technique.

### Comparison of associated complications

At the last follow-up, there was no significant difference between the group A and the group B patients in the proportion of coronal imbalance, thoracic kyphosis abnormality, and PJK, but the proportion of sagittal imbalance and abnormal internal fixation in group A was significantly higher than in group B. It can be seen that a convex side block combined with concave side single growing rod technique is worse than the traditional bilateral growing rod technique for controlling the sagittal balance of the spine. From a mechanical view this may be related to a single rod having less control of the overall torsion of the spine than does a double rod, and therefore a higher risk of internal fixation related complications. Our experience is that when there is a broken rod or pedicle screw is abnormal (the screw is obviously pulled out to cause spinal column imbalance, spinal cord nerve injury, etc.), the replacement with a bilateral growth rod technique is to be used for children who are still young and still have growth potential, while the original convex pedicle screw and short rod are removed. For children with spinal stiffness, multiple lengthenings, older and mature bone age, the rod is removed directly, the pedicle screw is replaced, and the final fusion internal fixation is performed [10]. Current follow-up results show the sagittal imbalance ratio and internal fixation abnormality ratio were significantly higher in group A than in group B. However, this is not to say that convex side short fusion combined with a concave side single growing rod technique cannot be used in clinical practice; it can still be used as an alternative to the traditional bilateral growing rod technique. For example, for children with thin back skin and soft tissue, it can be used as a surgical option. After the skin and soft tissue conditions on the back are improved, it can be changed to traditional bilateral growing rod technique if necessary.

Although some studies have shown that bilateral growing rods have fewer complications in the treatment of EOS than unilateral growing rod [7, 11], for some special types of EOS, there are reports that conclude that a unilateral growing rod technique can be considered. This would include special types of EOS such as complex underlying common diseases, determination of near-term final fusion, severe eczema, low body mass index, etc [12]. . . According to a similar principle, 18 EOS cases were treated with a single magnetically controlled growing rod combined with an apical vertebral block, with an average follow-up of 3-years, and the complications were lower than those of bilateral magnetically controlled growing rods [13]. The current trend is to use bilateral growing rod technology as far as is feasible. Regarding the PJK phenomenon after EOS treatment with the growing rod technique, some studies have pointed out that the incidence of PJK is 28%, among which the independent risk factor is that the upper end anchor is selected in the T2 vertebral [14]. Clinically, we found that the aggravation of thoracic kyphosis or the loss of kyphosis orthosis often led to the occurrence of PJK, which will lead to the loosening and displacement of the top anchor fixed point screw. In a multicenter EOS study with 34 patients treated with the growing rod technique, it was noted that repeated spinal bending exercise, syndrome-type EOS, and weight gain during treatment are risk factors for rod breakage, and the root cause is that repeated bending stress causes metal fatigue fracture of the rod. Frequent replacement of a rod is recommended during rod lengthening [15]. We believe that frequent rod replacement costs more, increases the surgical incision for each open operation, and increases the risk of wound infection, which is a trade-off problem. The optimal frequency of rod replacement remains to be further studied.

This study still has the following shortcomings: This study is a single-center study, although minimizing the variability of surgical techniques, the sample size included was small and the follow-up time was short, and not all patients underwent terminal fusion or terminated growth rod treatment.

## Conclusion

In the treatment of EOS, both convex side block combined with concave side single growing rod technique and the traditional bilateral growing rod technique can correct the main curve and allow the longitudinal growth of the spine. However, the traditional bilateral growth rod technique has more advantages in controlling sagittal balance of spine than convex side block combined with concave side single growing rod technique, and the complications associated with internal fixation are lower.

## Data Availability

The datasets supporting the conclusions of this article are included within the article.

## Data availability

The datasets used and/or analysed during the current study available from the corresponding author on reasonable request.

## Abbreviations

EOS: Early-onset scoliosis
SVA: Sagittal vertical axis
C7PL: C7 plumb line
CSVL: Center sacral vertical line
PJA: Proximal junctional angle
PJK: Proximal junctional kyphosis
